# Multifocal Analysis of Acute Pain After Third Molar Removal

**DOI:** 10.3389/fphar.2021.643874

**Published:** 2021-04-15

**Authors:** Giovana Maria Weckwerth, Thiago José Dionísio, Yuri Martins Costa, Paulo Zupelari-Gonçalves, Gabriela Moraes Oliveira, Elza Araújo Torres, Leonardo Rigoldi Bonjardim, Flavio Augusto Cardoso Faria, Adriana Maria Calvo, Troy Moore, Devin Michael Absher, Carlos Ferreira Santos

**Affiliations:** ^1^ Department of Biological Sciences, Bauru School of Dentistry, University of São Paulo, Bauru, Brazil; ^2^ Department of Biosciences, Piracicaba Dental School, University of Campinas, Piracicaba, Brazil; ^3^ Kailos Genetics Inc., HudsonAlpha Institute for Biotechnology, Huntsville, AL, United States; ^4^ HudsonAlpha Institute for Biotechnology, Huntsville, AL, United States

**Keywords:** catechol O-methyltransferase (COMT), opioid receptor, pain, NSAIDs (non-steroidal anti-inflammatory drugs), polymorphisms

## Abstract

**Background:** To analyze the pain modulation capacity profile in a Brazilian population, the relationship between opioid receptor (*OPRM1*) and Catechol-O-methyltransferase (*COMT*) 1polymorphisms and pain modulation capacity was determined through preoperative pain modulation tests and acute postoperative pain control evaluation, swelling, and trismus in 200 volunteers undergoing lower third molar removal.

**Methods:** Psychologic and clinical parameters were measured. Patient DNA was sequenced for single nucleotide polymorphisms in *OPRM1* and *COMT*, and the salivary concentration of interleukin (IL)-2 (IL)-6, interferon (IFN)-γ and tumor necrosis factor (TNF)-α was evaluated. Primary outcomes were the influence of all predictors on the fluctuation of pain intensity using a visual analogue scale (VAS), and swelling and trismus on the 2nd and 7th postoperative days. Preoperative pain modulation capacity (CPM), pain catastrophizing scale (PCS), body mass index (BMI), and surgery duration and difficulty were evaluated.

**Results:** Salivary concentration of IFN-γ and IL-2 as well as the duration of surgery influenced the fluctuation of postoperative pain in the VAS, and in the sum of the differences in pain intensity test at 8, 48, and 96 h. BMI influenced swelling, while both BMI and *COMT* haplotype influenced trismus on the 2nd postoperative day.

**Conclusion:** Polymorphisms in *COMT*, salivary concentrations of IL-2 and IFN-γ, BMI, and duration of surgery were predictors for pain fluctuation, swelling, and trismus on the 2nd day after lower third molar extraction. This therapy was effective in controlling inflammatory symptomatology after lower third molar extraction and ibuprofen was well tolerated by patients.

**Clinical Trial Registration:** www.ClinicalTrials.gov, identifier NCT03169127.

## Introduction

Classes of nociceptive pain include neuropathic, inflammatory and pathological (Merksey and Bogduk, 1994) pains. Different stimuli induce diverse physiological responses, and differences in genetic variability likely affect pain tolerance thresholds and levels (Fillingim et al., 2005; Crist and Berrettini, 2014).

Painful stimuli induce release of endogenous opioids, including endorphins, which activate opioid receptors (OPRM1) causing analgesic responses (Crist and Berrettini, 2014). The three most common opioid receptors are receptor µ-opioid (MOR), δ-opioid (DOR) and κ-opioid (KOR), encoded by the *OPRM1*, *OPRD1* and *OPRK1* genes, respectively (Crist and Berrettini, 2014). MOR is activated by endomorphins and β-endorphins (Crist and Berrettini, 2014).

Sequencing of several ethnic groups identified 3,324 polymorphisms in *OPRM1*. The most common single nucleotide polymorphism (SNP) in *OPRM1* is rs1799971 (Asn^40^Asp), referred to as A118G. This polymorphism has an overall frequency of 19% (Matsunaga et al., 2009; Crist and Berrettini, 2014) and is associated with functional effects (Crist and Berrettini, 2014). G allele carriers show behavioral differences in β-endorphin-mediated responses, including analgesia, euphoria, and sedation due to their 3-fold stronger binding (Bond et al., 1998; Chou et al., 2006; Lötsch et al., 2006; Matsunaga et al., 2009). A prevalence of 31.3% AA, 58.3% AG and 10.4% GG genotypes has been identified (Liu and Wang, 2012).

Investigators observed that serum concentrations of interleukin (IL)-6, tumor necrosis factor (TNF)-α and interferon (IFN)-γ are significantly lower and a quality of life health score is significantly higher in G allele carriers compared to subjects without allele G (Matsunaga et al., 2009). The endogenous opioid system is important in these carriers and may suppress proinflammatory cytokine secretion, influencing their health perception (Matsunaga et al., 2009).

The underpinnings of the descending pain inhibition also involve the central catecholaminergic systems, noradrenaline and dopamine (Jensen et al., 2009). This system is influenced by the catecholamine enzyme, encoded by catechol-O-methyltransferase (*COMT*), which moderates pain signal transmission through inactivation of catechol (dopamine, epinephrine, and norepinephrine) (Senagore et al., 2017). A highly studied SNP occurs in the coding region (rs4680G4A or Val^158^Met), and results in three possible genotypes [Met/Met (AA), Met/Val (AG), and Val/Val (GG)] (Jensen et al., 2009; Senagore et al., 2017). Reduction of *COMT* activity is related to increased pain sensitivity and the production of proinflammatory cytokines (Senagore et al., 2017). A recent systematic review showed that genetic polymorphisms of the catecholaminergic pathways are associated with thermal and blunt pressure sensitivity (Soares et al., 2020).

The conditioned pain modulation (CPM) paradigm is a valid and reliable psychophysical approach to evaluate the efficacy of the descending pain inhibition, under the assumption of “pain inhibits pain” that is a phenomenon that has important clinical applications, particularly, because can depict possible pain mechanisms underlying chronic pain conditions (Yarnitsky, 2015; Costa et al., 2017). CPM paradigms have also been reported as a potential tool for additional understanding of pain mechanisms that would improve treatment outcomes and can be held in a sequential form or parallel form (Costa et al., 2017; Soares et al., 2020), and in the present work we used the sequential CPM.

Lower third molar extraction model, in addition to allowing the evaluation of the efficacy of analgesic and anti-inflammatory drugs, enables the study of acute pain seen from other important angles that can contribute to the understanding of this multifactorial phenomenon called pain. (Cooper and Beaver, 1976; Akbulut et al., 2014; Silva de Oliveira et al., 2016; Degirmenci and Yalcin, 2019; Weckwerth et al., 2020).

Therefore, the primary aim of this study was to evaluate the predictive value of *OPRM1* and *COMT* polymorphisms, the preoperative proinflammatory cytokines on postoperative pain intensity, the pain modulation, the catastrophizing variables, swelling and trismus in individuals who underwent lower third molar removal. We hypothesized that all of the aforementioned variables contribute to the perception of pain in volunteers undergoing lower third molar removal.

## Materials and Methods

### Registration and Study Design

This study was performed in accordance with the Declaration of Helsinki and since this project involved human genetics, this study was evaluated and approved by the Institutional Ethics Committee of the Bauru School of Dentistry, University of São Paulo, and by the National Commission of Ethics Research (CONEP), Brazilian National Research Ethics System (CAAE number: 59807716.9.0000.5417), in accordance with resolution 466/12 of the National Council of the Ministry of Health, and registered at ClinicalTrials.gov ID (NCT03169127). All volunteers read and signed an Informed Consent form during screening prior to carrying out any study procedures. The sample size comprised 200 volunteers, and the sample calculation was obtained using a G*Power software v.3.0.10. The sample size calculation was performed based on swelling data from a previous study (Weckwerth et al., 2016). The sample size should be composed by 193 patients, with minimal effect size of 0.18, power of 70% and significance level of 5%.

Briefly, 210 adults (≥18 years old) out of 300 were selected to participate in this study of unilateral lower third molar extraction after analysis of their panoramic radiograph (Weckwerth et al., 2016; Simoneti et al., 2018; Weckwerth et al., 2020), according to dental indications, such as orthodontic, endodontic and periodontal problems. All volunteers should have similar lower third molar position and similar degree of impaction according to Pell and Gregory’s classification in order to provide similar tissue trauma during surgery (Simoneti et al., 2018); this information can be assessed on Table 1. Additionally, per the inclusion criteria, volunteers could not have systemic diseases, which could interfere in the study, and the extraction sites could not have inflammation or infection.

**TABLE 1:** Preoperative, intraoperative and postoperative parameters.

Parameter	Unit
Gender	*N* and %
Age	(Mean years. SD)
BMI	kg/Height^2^
OPRM1 haplotypes	Haplotype mutated or ancestral
COMT haplotypes	Haplotype mutated or ancestral
Conditioned pain modulation (CPM)	Pressure pain threshold (PPT)—%
Pain catastrophizing scale (PCD)	Range from 0 to 52 points
Lower third molar position	Pell and gregory classification (IA, IB, IC, IIA, IIB, IIC, IIIA, IIIB, IIIC)
Surgery difficulty—(score assessed by surgeon)	3-Point scale: 1) no need for osteotomies without tooth sectioning; 2) need for osteotomies without tooth sectioning; 3) need for osteotomies and tooth sectioning complicated
Mouth opening	Preoperative period, 2nd, 7th postoperative day (mm)
Facial swelling	Preoperative period, 2nd, 7th postoperative day (mm)
Subjective evaluation of postoperative pain	Visual analog scale (VAS, 0–100 mm)
Overall experience of surgery reported by volunteer in the 7th postoperative day	5-Point scale: 1) “poor”; 2) “fair”; 3) “good”; 4) “very good”; 5) “excellent”

**FIGURE 1:**
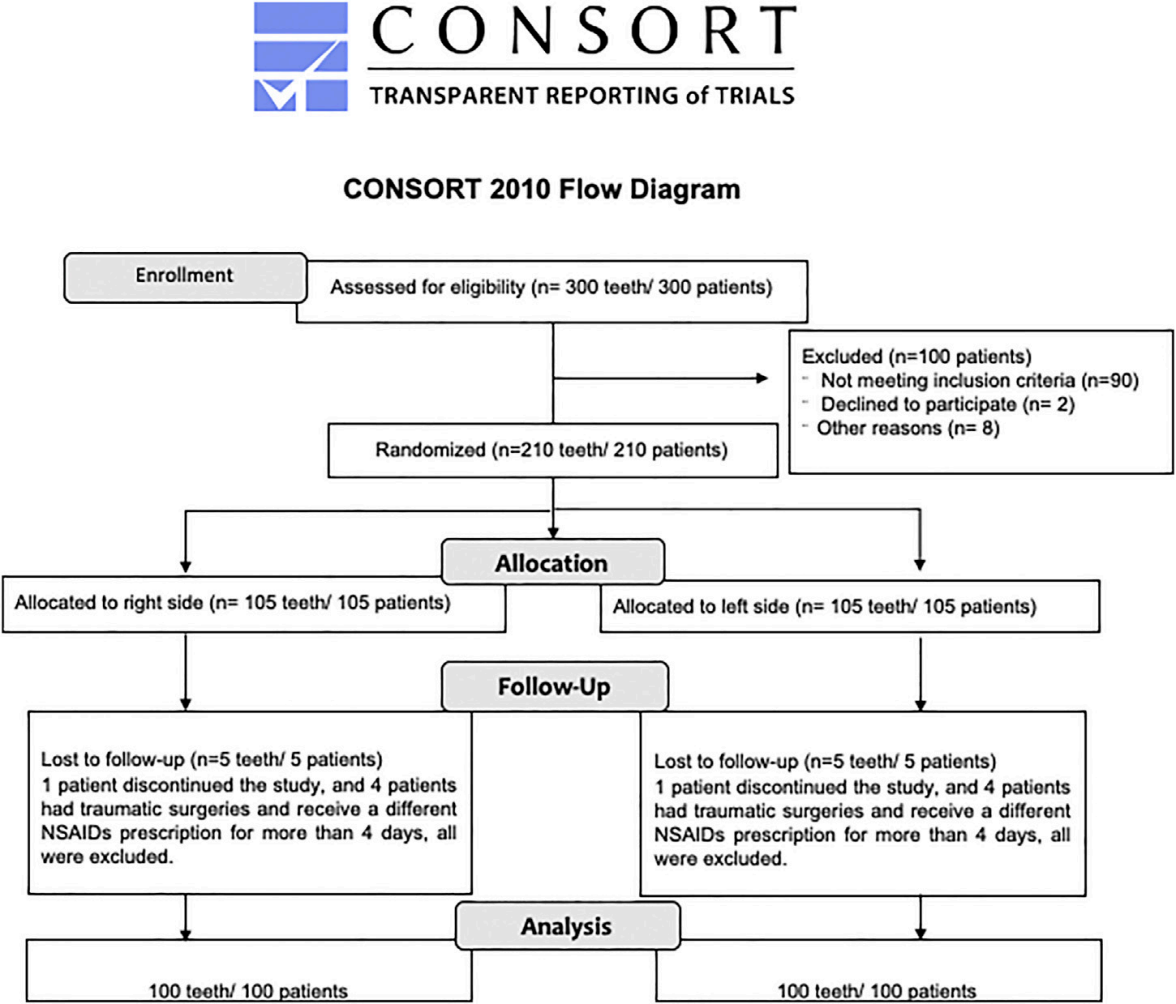
Consort flow diagram (Weckwerth et al., 2020).

Volunteers were excluded from the study if they presented a history of allergy to local anesthetics, kidney disease, history of bleeding or gastrointestinal ulcers, asthma, hepatic, kidney, intestinal, cardiac, pulmonary, circulatory and/or brain dysfunction or allergic sensitivity to any NSAIDs. Volunteers that were pregnant or breast-feeding, and individuals that used antidepressants, anticoagulants, diuretics and/or antibiotics within 2 months before surgery, and with chronic pain confirmed during the anamnesis, were also excluded (Santos et al., 2007; Senes et al., 2015; Calvo et al., 2017; Zupelari-Goncalves et al., 2017). Thus, 90 patients out of 300 were excluded since they did not meet these criteria and 210 patients were submitted to lower third molar extraction. Specifically, eight volunteers who had very traumatic surgeries and needed to use higher doses of local anesthetics and NSAIDs were excluded from the study and two other volunteers dropped out from the study (Figure 1).

### Surgery Intervention and Assessments

At the end of this study a total of 200 extracted lower third molars from 200 volunteers were analyzed. Initially, 210 volunteers with the dental indication to remove at least one lower third molar were screened for participation, after completing all inclusion criteria. Of these 210 volunteers, ten were excluded from the study. Two dropped out of the study and the other eight volunteers experienced very traumatic surgeries, and required increased NSAID dosage. Of the eight excluded volunteers, two experienced postoperative infection on the extraction site and received antibiotic therapy.

All costs of this research were funded by the São Paulo Research Foundation (FAPESP—Brazil). Dental surgeries were performed from December 2016 through July 2018 at the Clinical of Pharmacology and Physiology Laboratory (LAFFIC) at the Bauru School of Dentistry (FOB), University of São Paulo (USP), Bauru, SP, Brazil, by the same dental surgeon (GMW), using a worldwide standardized (Trindade et al., 2011; Calvo et al., 2012; Weckwerth et al., 2016; Zupelari-Goncalves et al., 2017; Weckwerth et al., 2020), in which the amount of the local anesthetic used (4% articaine with 1:200,000 epinephrine), the duration and the surgical trauma of the operated volunteers were standardized. The side to be operated (right or left) was determined randomly (http://www.randomization.com, number: 20434). Surgical parameters evaluated during and after the surgeries analyzed in this study are described in Table 2. Patients who had another lower third molar with an indication for extraction were operated on approximately 1 month after the first surgery. This second procedure was held according to the same surgical protocol and the data were analyzed as part of another research by this same group, titled “Influence of the cytochrome P450 (CYP2C9) genotype on clinical efficacy of tenoxicam,” assessed and approved by the Institutional Ethics Committee of the Bauru School of Dentistry, University of São Paulo (CAAE number: 66699717.3.1001.5420) and registered at ClinicalTrials.gov ID (NCT04182191).

**TABLE 2:** Evaluated study parameters.

Parameters evaluated	All (*n* = 200)	
Parameter (*n*, %)	*N*	%
Male (*n*, %)	67	33.5
Female (*n*, %)	133	66.5
Parameter (mean, SD)	Mean	SD
Age (mean years. SD)	24	6.5
BMI (mean kg/m^2^. SD)	25.17	5.57
Lower third molar position (pell and gregory classification; *n*. %)	*N*	%
IA	92.0	46.0
IB	27.0	13.5
IC	5.0	2.5
IIA	33.0	6.0
IIB	11.0	16.5
IIC	12.0	5.5
IIIA	1.0	7.5
IIIB	4.0	2.0
IIIC	15.0	0.5
Gene haplotypes (*n*)	Ancestral	Mutated
OPRM1 (five undetermined)	139	56
COMT (30 undetermined)	62	108
Modulation of pain tests (mean, SD)	Mean	SD
Conditioned pain modulation (CPM) (mean CPM. SD)	−67.97	45.3
Pain catastrophizing scale (PCS) (mean points.SD)	21.31	11.41
Surgery difficulty—(score assessed by surgeon) (*n*, %)	*N*	%
1) no need for osteotomies without tooth sectioning	5	2.5
2) need for osteotomies without tooth sectioning	101	50.5
3) need for osteotomies and tooth sectioning complicated	94	47.0
Mouth opening (median mm. IQR)	Median	IQR
Preoperative measurement	50.0	15.0
2nd day postoperative measurement	25.0	10.0
7th day postoperative measurement	34.0	15.0
Facial swelling (mean mm. SD)	Mean	SD
Preoperative measurement	370.0	30.0
2nd day postoperative measurement	387.0	30.0
7th day postoperative measurement	380.0	30.0

For the complete local anesthesia, a block of the inferior alveolar, oral, and lingual nerves, using a single 1.8-ml cartridge of anesthetic was performed. An additional 0.9 ml of local anesthetic (half of a cartridge) was administered around the soft tissue of lower third molar to reduce bleeding and ensure mucosal anesthesia (Zupelari-Goncalves et al., 2017). After anesthesia block, the surgical procedure was started (Calvo et al., 2012; Weckwerth et al., 2016; Zupelari-Goncalves et al., 2017; Simoneti et al., 2018) and surgeries that were very traumatic due the quantity of osteotomies and tooth sectioning, with duration of more than 40 min and additional complementation of anesthesia were excluded of the study since these patients could present more pain, swelling and trismus than the other patients. Hence, these were excluded from the study and received different dosage of medication.

For pain and inflammation control, one tablet of 600 mg ibuprofen was consumed by volunteers every 8 h for 4 days; the first tablet was taken immediately after the procedure (Troullos et al., 1990; de Oliveira et al., 2016). Rescue medication (750 mg acetaminophen tablets) was also provided; volunteers could consume this medication to supplement the ibuprofen effect every 8 h, if they fell the necessity.

Evaluation of postoperative pain was determined using a visual analogue scale (VAS, 0–100 mm) with 0 mm indicating no pain and 100 mm indicating the worst possible pain, as previously described (Trindade et al., 2012; Simoneti et al., 2018). Volunteers recorded their postoperative pain using the VAS at the following time points after the surgical procedure: 0, 0.25, 0.5, 0.75, 1, 1.5, 2, 3, 4, 5, 6, 7, 8, 10, 12, 18, 24, 48, 72, and 96 h (0 = immediately after the surgery). Volunteers also received a VAS form to record postoperative pain experienced when they used the rescue medication. The principal investigator (GMW) evaluated the swelling, trismus and temperature at pre, intra and postoperative periods.

Using the method proposed by Üstün and colleagues (2003), three facial measures (A, B and C) were obtained with flexible measuring tape. Preoperative sum of the three measurements was considered baseline value. Differences obtained between baseline and postoperative values indicated facial swelling on the 2nd and 7th postoperative days (Weckwerth et al., 2016; Zupelari-Goncalves et al., 2017; Simoneti et al., 2018).

Quantity of mouth opening (trismus), also obtained with a flexible tape, was considered by measuring the distance (in mm) between the edges of incisors during mouth opening before on the 2nd and 7th postoperative days (Weckwerth et al., 2016; Zupelari-Goncalves et al., 2017; Simoneti et al., 2018).

### Genetic Sequencing and Analyses

Briefly, the saliva of the 200 volunteers was previously collected. Genomic DNA was extracted using the QIAamp DNA Mini Kit (Cat No./ID: 51306, QIAGEN^®^ Hilden, Germany) and lyophilized with the FreeZone 4.5 L Benchtop Freeze Dry System (Catalog #: 7750020, Labconco^®^, Kansas City, MO, United States). Lyophilized DNA was utilized for genetic sequencing of *OPRM1* rs1799971 and *COMT* rs4680 using MiSeq^®^ System (Illumina^®^, San Diego, CA, United States) instruments with a 2 bp × 78 bp read length, and the protocol of Kailos Genetics Inc. (Huntsville, AL, United States).

### MILLIPLEX^®^ Enzyme-Linked Immunosorbent Assay (ELISA) Detection of Proinflammatory Cytokines in Saliva

Saliva samples of the 200 volunteers were collected 30 min before the surgical procedure. MILLIPLEX^®^ ELISA was used to detect the presence of four proinflammatory cytokines (IL-2, IL-6, IFN-γ and TNF-α) using the HCYTOMAG-60K MILLIPLEX ELISA kit (Milliplex MAP Human Cytokine/Chemokine Kit; Millipore, Billerica, MA, United States), according to the manufacturer’s protocol.

### Conditioned Pain Modulation and Pain Catastrophizing Scale

In order to check the functioning of this group of neurons, the conditioned pain modulation paradigm (CPM) was utilized. CPM has important clinical applications (Yarnitsky, 2015; Costa et al., 2017). This test was applied to assess if patients’ pre surgical pressure pain scores had any correlation with their postsurgical pain. Prior to surgery, all volunteers underwent the protocol of CPM sequential test. Briefly, the pressure pain threshold (PPT) was utilized as a test stimulus (TS) and the conditioning stimulus (CS) was the submersion of the non-dominant hand in a 46°C water bath for 1 min. PPT was measured by means of a 1 cm^2^ flat circular tip algometer with constant and increasing pressure application of 0.5 kg/cm^2^/s in the anterior temporal muscle region of the dominant side. TS was measured before and after CS. PPT was calculated by subtracting the absolute values of TS after CS minus TS before CS; the test was measured three times, then the mean was obtained and the relative change was considered as a percentage (Yarnitsky, 2015; Porporatti et al., 2017).

Volunteers also answered a questionnaire that measures catastrophic thoughts in pain, the PCS (Sullivan et al., 1995), that has been translated and validated in Portuguese (Sehn et al., 2012). Before the surgery, volunteers answered 13 questions that indicated the frequency of catastrophic thoughts when they feel strong pain. This frequency scale ranged from 0 to 5 (0 = almost never and 5 = almost always), and the total score was calculated by summing all items, ranging from 0 to 52 points (Sehn et al., 2012).

## Statistical Analysis

### Data Collected and Surgical Outcomes

Data were analyzed using Microsoft^®^ Excel 2002 (version 10.6871.6870) and Graph Pad Prism (version 8.3.1). Description of quantitative data is presented as the mean and standard deviation (SD), while qualitative variables are presented as a percentage. For the inferential analysis, Shapiro-Wilk test was performed to verify the normality in the distribution of quantitative variables.

Multiple linear regression models were used to determine the main factors associated with the postoperative pain intensity, swelling and trismus. Therefore, the dependent variables analyzed were: pain intensity at times of 8, 48, and 96 h after surgery as well as swelling and trismus. This study used a time variable, the sum of the pain intensity (SPI), that is the value obtained by summing the scores of individual pain intensity from the baseline at each assessment time (Cooper et al., 2016). The independent variables were: CPM magnitude, PCS, surgery difficulty and duration, IL-2, IL-6, INF-γ, and TNF-α concentrations, body mass index (BMI), and the genetic profile of *OPRM1* and *COMT* haplotypes. SPI calculation was used in pain analyses, and an ANOVA was used to verify the relevance of the SPI. Statistical significance was set at 0.05.

## Results

A total of 200 volunteers (200 lower third molars) were studied, of which 133 (66.5%) were from female volunteers and 67 (33.5%) were from male volunteers (Table 1). The mean age (SD) of the volunteers was 24 (6.5) years, with an age range of 18–57 years. BMI was calculated by the division of kilograms by squared height of each volunteer; the mean of all volunteers was 25.17 kg/m^2^ (5.57 SD) (Table 1).

According to the CPM test, the magnitude of the preoperative CPM was −67.97% (45.3 SD). The average frequency of catastrophic thoughts was 21.31 points in the 200 volunteers, after answering the questions from the PCS (Table 1).

Genetic sequencing results related to *COMT* and *OPRM1* haplotypes, obtained using the MiSeq^®^ Illumina^®^ instrument, are described in Table 3. For the *OPRM1* haplotypes, 69.5% of volunteers were A/A, characterized as a normal metabolizer, 25.5% were identified as A/G, reduced metabolizers, and 2.5% were G/G, characterized as poor metabolizers. For the *COMT* haplotypes, 31% of volunteers were G/G, characterized as normal metabolizers, 38% were A/G, reduced metabolizers, and 16% were identified as A/A, poor metabolizers. During genetic sequencing, the *OPRM1* and *COMT* haplotypes of 2.5 and 15% of this population, respectively, could not be determined due the DNA quality during the processing (Table 3).

**TABLE 3:** Percentage and number of volunteers that presented mutations in the opioid receptor (*OPRM1*) and catechol-O-methyltransferase (*COMT*) genes.

OPRM1 haplotypes	% (*n*)	Type of metabolizer	COMT Haplotypes	% (*n*)	Type of metabolizer
A/A	69.5% (139)	Normal	G/G	31% (62)	Normal
A/G	25.5% (51)	Reduced	A/G	38% (76)	Reduced
G/G	2.5% (5)	Poor	A/A	16% (32)	Poor
Indeterminate	2.5% (5)	—	Indeterminate	15% (30)	—

In the multiple linear regressions performed with all studied variables, the *OPRM1* and *COMT* haplotypes were divided into two groups of Mutated and Ancestral. Thus, patients that were considered reduced and poor metabolizers were grouped and analyzed as Mutated, and the normal metabolizers were analyzed as Ancestral (Table 1).

SPI calculation was performed to evaluate which type of variable influenced the fluctuation of reported postoperative pain scores in the VAS during all analyzed periods. A multiple linear regression was used to predict SPI from the concentration of IL-2, IL-6, IFN-γ and TNF-α, the *OPRM1* and *COMT* haplotypes, BMI, CPM, PCS, and surgery duration and difficulty. The multiple regression model was significant (*p* = 0.2617) with an *R* ^2^ of 0.08072. Two predictors contributed significantly to this result, IL-2 (*p* = 0.046) and duration of surgery (*p* = 0.0157) (Supplementary Table S1). Likewise, considering pain at the period of 8 h after surgery as the dependent variable and using the same eleven independent variables, three variables added significantly to the prediction (*p* = 0.1736): the presence of IFN-γ (*p* = 0.0248), IL-2 (*p* = 0.0176) and the duration of surgery (*p* = 0.0228) (*R* ^2^ = 0.09092). Regression coefficients and standard errors are found in Supplementary Table S2.

Another multiple linear regression was used to predict pain at 48 h after surgery, using the same independent variables (*p* = 0.1597), and again, the predictors IL-2 (*p* = 0.0107) and duration of surgery (*p* = 0.0142) influenced the pain fluctuation in the VAS (*R* ^2^ = 0.09223) (Supplementary Table S3). Using the same model, at 96 h after the procedure, only duration of surgery (*p* = 0.007) was a predictor of pain fluctuation, with *R* ^2^ = 0.09198, and a significance of *p* = 0.1615 (Supplementary Table S4).

This regression model also evaluated which variables influenced the quantity of swelling on the 2nd postoperative day, and between all variables analyzed (IL-2, IL-6, INF-γ, and TNF-α, the *OPRM1* and *COMT* haplotypes, BMI, CPM, PCS, and surgery duration and difficulty), only the model containing BMI was a predictor for swelling on the 2nd postoperative day (*p* = 0.0177, *R* ^2^ = 0.0855) (Supplementary Table S5). An ANOVA test confirmed this result.

One last linear regression analysis was performed to evaluate the influence of these variables on the presence of trismus (related to the mouth opening) on the 2nd postoperative day. When all variables were analyzed, the presence of the *COMT* haplotype (*p* = 0.0119) and BMI (*p* = 0.029) predicted the presence of trismus (*p* = 0.0385, *R* ^2^ = 0.1202); an ANOVA test confirmed the relevance of this model (Supplementary Table S6).

## Discussion

In this study multiple linear regression was performed using data from 200 patients who underwent lower third molar extraction with ibuprofen therapy to evaluate if the fluctuation of postoperative pain (determined by the VAS) and the quantity of swelling and trismus on the 2nd postoperative day were influenced or predicted by the following variables: concentration of IL-2, IL-6, IFN-γ, and TNF-α; *OPRM1* and *COMT* haplotypes, BMI, CPM, PCS, surgery duration, and difficulty. The main findings were: 1) The concentration of two proinflammatory cytokines, IFN-γ and IL-2, and the duration of surgery were the principal predictors that influenced the fluctuation of postoperative pain in the VAS and in the SPI calculation at 8, 48, and 96 h. 2) BMI influenced both the presence of swelling and trismus on the 2nd postoperative day. 3) *COMT* haplotype also influenced the presence of trismus on the 2nd postoperative day.

This study is based on the hypothesis that patients with polymorphisms in *OPRM1* and *COMT* have differences in the levels of perception and modulation of pain, since carriers of these polymorphisms exhibit different affinities for the binding sites of their receptors, which determines different analgesic capacities (Bartošová et al., 2015), according to the endogenous ligand that is coupled to these receptors. Therefore, people with these polymorphisms may have different perceptions of pain when treated with opioids. The most common SNP studied in *OPRM1* is rs1799971, referred to as A118G (Matsunaga et al., 2009; Crist and Berrettini, 2014). When treated with opioids, or rescue medication, individuals with the G allele have more pain symptoms than individuals without the G allele, and thus have increased adverse effects, such as those demonstrated in studies evaluating orthopedic surgeries (Bond et al., 1998; Bartošová et al., 2015). In our study, the frequency of *OPRM1* haplotypes was similar to the ratio reported previously, where, among the 200 volunteers, 69.5% were A/A, 25.5% were A/G and 2.5% were G/G (Trescot et al., 2008; Liu and Wang, 2012). However, in the present study we did not observe a relationship between the effectiveness of a NSAID with the rs1799971 genotype and the control of acute postoperative pain by VAS, which demonstrated that this population does not have different analgesic capacities, even in those with the G allele, which could possibly present some differences in the modulatory capacity of pain mediated by endogenous mechanisms. These results contrast with other studies that found a relationship for *OPRM1* polymorphisms, mainly in carriers of the variant 118G allele, and postoperative pain after opioids treatment (Bartošová et al., 2015), because these patients required higher opioid doses for pain relief (Zhang et al., 2005; Chou et al., 2006).

The presence of the proinflammatory cytokines IFN-γ and IL-2 as well as the duration of surgery were important predictors for the fluctuation of postoperative pain in the VAS and the SPI calculation at 8, 48, and 96 h after surgery (Supplementary Table S2). The endogenous opioid peptide β-endorphin is known to regulate secretion of proinflammatory cytokines from peripheral immune cells through mechanisms dependent on the µ-opioid receptor, including IL-2, IL-6, TNF-α, and IFN-γ. Matsunaga and collaborators found a strong relationship between decreased concentrations of cytokines and a higher quality of life assessed by G allele carriers compared to individuals without the G allele (Matsunaga et al., 2009). Thus, it is expected that G allele carriers have different amounts of circulating cytokines, which influence their perception and postoperative pain modulation (Matsunaga et al., 2009). In the present study, it was observed that individuals with increased salivary concentration of IL-2 and IFN-γ demonstrated increased pain fluctuation in the SPI calculation, 8 and 48 h after surgery, than individuals with decreased concentrations. Thus, we infer that the concentrations of IL-2 and IFN-γ influenced the fluctuation of acute postoperative pain in this Brazilian population.

The duration of surgery was a significant predictor in the multiple linear regression analysis and influenced the fluctuation of postoperative pain in the SPI calculation at 8, 48, and 96 h after surgery (Supplementary Tables S1–S4). An important finding of the present research was that the duration of surgery probably affected tissue trauma, suggesting that a longer time of tissue manipulation generates increased tissue trauma (Troullos et al., 1990; Graziani et al., 2006), which influences the intensity of swelling and inflammation after surgery. We hypothesize that, consequently, there were increased levels of inflammatory infiltrates containing proinflammatory cytokines around the manipulated area after surgery, which presents a potential for pain that influences the amount of postoperative pain (Theken et al., 2019). The duration of surgery is strongly correlated with tooth position, the degree of surgical trauma, the need for removing bone tissue, complications in the intraoperative period, the extension of the flap, and periosteum displacement around the surgical site, which influences postoperative pain (Antunes et al., 2011).

Another predictor that influenced both swelling and trismus on the 2nd postoperative day in the present study was BMI (Supplementary Tables S5, S6). Volunteers with lower BMI showed increased mouth opening limitation (trismus) while swelling was greater in volunteers with higher BMI. This index is measured by the correlation between weight and height that determines body fat, and has been widely described in previous studies (Santana-Santos et al., 2013; Pérez-González et al., 2018). Pérez-González and collaborators found in their research that BMI and other predictors (gender, relation to lingual and buccal walls, and age) are determinants in explaining swelling. Specifically, BMI has a low influence on swelling in their patients, and trismus is not influenced by these predictors (Pérez-González et al., 2018), which is contrasting result of the present research. Obesity contributes to systemic inflammation, since the adipose tissue increases the expression and secretion of TNF-α, IL-6 and other proinflammatory cytokines involved in inflammation (Ferrante, 2007; Pérez-González et al., 2018). The surveyed patients with a higher BMI do not have significant differences in the level of interleukins measured before surgery in the present study (data not shown), but these patients had more facial swelling on the 2nd postoperative day without, however, influencing the amount of mouth opening. On the contrary, patients with lower BMI presented trismus, which can be explained by the degree of inflammation of the muscles and tissues around the operated area, which may have influenced the increased mouth opening limitation observed on the 2nd postoperative day.

Catechol-O-methyltransferase encoded by *COMT* is another important and well-studied enzyme related to transmission of pain signals in the body (Martire et al., 2016; Senagore et al., 2017), and is correlated with variations of pain sensitivity in experimental models of noxious stimuli (Kim et al., 2006; Lee et al., 2011). The most studied SNP is rs4680G4A, recognized as Val^158^Met (Kim et al., 2006; Martire et al., 2016; Senagore et al., 2017). Increased pain sensitivity has been correlated with the production of proinflammatory cytokines in patients with *COMT* haplotypes with reduced enzymatic activity (Senagore et al., 2017). In the 200 patients of the present study, the prevalence was 31% (GG), 38% (AG) and 16% (AA), similar to that found in the literature (Martire et al., 2016).

We did not find an association between *COMT* SNP rs4680 and acute postoperative pain after lower third molar extraction, as observed in another survey, albeit with a weak relationship (Kim et al., 2006). An important finding of the present study was that the *COMT* mutated allele (AG and GG genotypes) influenced trismus on the 2nd postoperative day (Supplementary Table S6). Patients with these genotypes showed greater mouth opening limitation (trismus) on the 2nd postoperative day when compared to the pre-operative measures observed before surgery.

In this research, the frequency of catastrophic thoughts (21.31) and the magnitude of pain modulation assessed by the CPM test [−67.97% (45.3 SD)] were not predictors capable of strongly leading to postsurgical pain assessed by the visual analogue scale. CPM value was used, which is a test that assesses the level and magnitude of conditioned pain modulation of patients, through test stimuli before and after the conditioning stimulus in order to verify if patients who have a greater pain modulation profile or less in the preoperative moment are able to modulate or influence the postoperative pain in a positive way or not (Costa et al., 2017; Porporatti et al., 2017), and such correlation was not found in the present study.

A limitation of the present study is that most studies discuss chronic and acute pain, including osteoarthritis of the knee (Martire et al., 2016), laparoscopic cholecystectomy pain (Jensen et al., 2005), and surgeries, such as total knee arthroplasty and others (Chou et al., 2006; Senagore et al., 2017). In addition, in such studies patients received opioids, such as morphine, piritramide, and fentanyl (Zhang et al., 2005; Chou et al., 2006; Bartošová et al., 2015). In our model, we evaluated patients after lower third molar extraction under NSAID therapy (ibuprofen). In the present study, postoperative cytokine concentrations were not evaluated and there was no control group that did not use NSAIDs but only analgesics.

## Conclusion

Polymorphisms in the *COMT* gene, the concentrations of IL-2 and IFN-γ cytokines in saliva, BMI and the duration of surgery were predictors for the pain perception and presence of swelling and trismus on the 2nd day after lower third molar extraction.

## Data Availability

The datasets presented in this study can be found in online repositories. The names of the repository/repositories and accession number(s) can be found in the article/Supplementary Material.
