# Supplementary material for: Multifocal Analysis of Acute Pain After Third Molar Removal
Source: Front Pharmacol. 2021 Apr 15;12:643874. doi: 10.3389/fphar.2021.643874 (PMC8082138; doi:10.3389/fphar.2021.643874)
Supplement: Supplementary file 1 [file table1.docx]

**Table S1 -** Multiple logistic regression model. The sum of pain intensity (SPI) (fluctuation of pain) is the dependent variable and interferon (IFN)-γ, interleukin (IL)-2, (IL)-6, tumor necrosis factor (TNF)-α, body mass index (BMI), surgery difficulty and duration, opioid receptor (*OPRM1)* and catechol-O-methyltransferase (*COMT)* haplotype, pain modulation capacity (CPM), and pain catastrophizing scale (PCS) are independent variables.

|  | **SPI** | | | | |
| --- | --- | --- | --- | --- | --- |
| **Variable** | **β** | **S. E** | **P value** | **β - 95% CI** |  |
| **Intercept** | -70 | 66.83 | 0.2965 | -202.0 to 62.00 |  |
| **IFN**-γ | 41.53 | 21.51 | 0.0553 | -0.9544 to 84.01 |  |
| **IL-2** | -105.7 | 52.52 | **0.046** | -209.4 to -1.922 |  |
| **IL-6** | -6.007 | 3.774 | 0.1135 | -13.46 to 1.448 |  |
| **TNF-**α | 3.01 | 2.931 | 0.3061 | -2.780 to 8.799 |  |
| **BMI** | -0.07904 | 1.716 | 0.9633 | -3.469 to 3.311 |  |
| **Surg. Difficult** | 37.94 | 22.28 | 0.0907 | -6.080 to 81.96 |  |
| **Surg. Duration** | -3.798 | 1.555 | **0.0157** | -6.869 to -0.7265 |  |
| **OPMR1** | 6.886 | 22.16 | 0.7564 | -36.88 to 50.65 |  |
| **COMT** | -23.95 | 20.46 | 0.2435 | -64.37 to 16.46 |  |
| **CPM** | -3.459 | 20.61 | 0.8669 | -44.16 to 37.25 |  |
| **PCS** | 0.7847 | 0.8617 | 0.3639 | -0.9173 to 2.487 |  |

Sum of in pain intensity (SPI),interferon (IFN)-γ, interleukin (IL)-2, (IL)-6, tumor necrosis factor (TNF)-α, body mass index (BMI), opioid receptor (*OPRM1)* and catechol-O-methyltransferase (*COMT)* haplotype, pain modulation capacity (CPM), pain catastrophizing scale (PCS), standard errors (S.E), confidence interval (CI).
