# Supplementary material for: Multifocal Analysis of Acute Pain After Third Molar Removal
Source: Front Pharmacol. 2021 Apr 15;12:643874. doi: 10.3389/fphar.2021.643874 (PMC8082138; doi:10.3389/fphar.2021.643874)
Supplement: Supplementary file 2 [file table2.docx]

**Table S2 -** Multiple logistic regression model. Pain, 8 h after surgery, is the dependent variable and interferon (IFN)-γ, interleukin (IL)-2, (IL)-6, tumor necrosis factor (TNF)-α, body mass index (BMI), surgery difficulty and duration, opioid receptor (*OPRM1)* and catechol-O-methyltransferase (*COMT)* haplotype, pain modulation capacity (CPM), and pain catastrophizing scale (PCS) are independent variables.

|  | **Pain after 8h** | | | | |
| --- | --- | --- | --- | --- | --- |
| **Variable** | **β** | **S. E** | **P value** | **β - 95% CI** |  |
| **Intercept** | 10.65 | 6.861 | 0.1225 | -2.899 to 24.21 |  |
| **IFN**-γ | -4.998 | 2.206 | **0.0248** | -9.356 to -0.6406 |  |
| **IL-2** | 12.92 | 5.385 | **0.0176** | 2.282 to 23.56 |  |
| **IL-6** | 0.5392 | 0.3871 | 0.1656 | -0.2254 to 1.304 |  |
| **TNF-**α | -0.2003 | 0.3005 | 0.506 | -0.7940 to 0.3933 |  |
| **BMI** | -0.2537 | 0.1762 | 0.152 | -0.6017 to 0.09440 |  |
| **Surg. Difficult** | -2.196 | 2.289 | 0.3387 | -6.717 to 2.324 |  |
| **Surg. Duration** | 0.3667 | 0.1594 | **0.0228** | 0.05181 to 0.6816 |  |
| **OPMR1** | 1.52 | 2.276 | 0.5052 | -2.976 to 6.016 |  |
| **COMT** | 2.674 | 2.099 | 0.2047 | -1.473 to 6.821 |  |
| **CPM** | -0.9902 | 2.113 | 0.64 | -5.164 to 3.183 |  |
| **PCS** | -0.02906 | 0.08877 | 0.7439 | -0.2044 to 0.1463 |  |

Interferon (IFN)-γ, interleukin (IL)-2, (IL)-6, tumor necrosis factor (TNF)-α, body mass index (BMI), opioid receptor (*OPRM1)* and catechol-O-methyltransferase (*COMT)* haplotype, pain modulation capacity (CPM), pain catastrophizing scale (PCS), standard errors (S.E), confidence interval (CI).
