# Supplementary material for: Multifocal Analysis of Acute Pain After Third Molar Removal
Source: Front Pharmacol. 2021 Apr 15;12:643874. doi: 10.3389/fphar.2021.643874 (PMC8082138; doi:10.3389/fphar.2021.643874)
Supplement: Supplementary file 3 [file table3.docx]

**Table S3-** Multiple logistic regression model. Pain, 48 h after surgery, is the dependent variable and interferon (IFN)-γ, interleukin (IL)-2, (IL)-6, tumor necrosis factor (TNF)-α, body mass index (BMI), surgery difficulty and duration, opioid receptor (*OPRM1)* and catechol-O-methyltransferase (*COMT)* haplotype, pain modulation capacity (CPM), and pain catastrophizing scale (PCS) are independent variables

|  | **Pain after 48h** | | | | |
| --- | --- | --- | --- | --- | --- |
| **Variable** | **β** | **S. E** | **P value** | **β - 95% CI** |  |
| **Intercept** | 2.211 | 5.431 | 0.6845 | -8.516 to 12.94 |  |
| **IFN**-γ | -3.289 | 1.748 | 0.0617 | -6.741 to 0.1627 |  |
| **IL-2** | 11.03 | 4.267 | **0.0107** | 2.597 to 19.46 |  |
| **IL-6** | 0.1277 | 0.3067 | 0.6777 | -0.4781 to 0.7335 |  |
| **TNF-**α | -0.02416 | 0.2382 | 0.9193 | -0.4946 to 0.4463 |  |
| **BMI** | -0.04694 | 0.1395 | 0.7369 | -0.3224 to 0.2285 |  |
| **Surg. Difficult** | -1.385 | 1.811 | 0.4457 | -4.962 to 2.192 |  |
| **Surg. Duration** | 0.3133 | 0.1263 | **0.0142** | 0.06369 to 0.5628 |  |
| **OPMR1** | -2.34 | 1.8 | 0.1956 | -5.896 to 1.216 |  |
| **COMT** | 0.9383 | 1.663 | 0.5733 | -2.346 to 4.222 |  |
| **CPM** | 0.9278 | 1.675 | 0.5803 | -2.380 to 4.235 |  |
| **PCS** | -0.01545 | 0.07002 | 0.8256 | -0.1538 to 0.1229 |  |

Interferon (IFN)-γ, interleukin (IL)-2, (IL)-6, tumor necrosis factor (TNF)-α, body mass index (BMI), opioid receptor (*OPRM1)* and catechol-O-methyltransferase (*COMT)* haplotype, pain modulation capacity (CPM), pain catastrophizing scale (PCS), standard errors (S.E), confidence interval (CI).
