# Supplementary material for: Multifocal Analysis of Acute Pain After Third Molar Removal
Source: Front Pharmacol. 2021 Apr 15;12:643874. doi: 10.3389/fphar.2021.643874 (PMC8082138; doi:10.3389/fphar.2021.643874)
Supplement: Supplementary file 4 [file table4.docx]

**Table S4 –** Multiple logistic regression model. Pain, 96 h after surgery, is the dependent variable and interferon (IFN)-γ, interleukin (IL)-2, (IL)-6, tumor necrosis factor (TNF)-α, body mass index (BMI), surgery difficulty and duration, opioid receptor (*OPRM1)* and catechol-O-methyltransferase (*COMT)* haplotype, pain modulation capacity (CPM), and pain catastrophizing scale (PCS) are independent variables

|  | **Pain after 96 h** | | | | |
| --- | --- | --- | --- | --- | --- |
| **Variable** | **β** | **S. E** | **P value** | **β - 95% CI** |  |
| **Intercept** | -0.1211 | 5.467 | 0.9824 | -10.92 to 10.68 |  |
| **IFN**-γ | -2.746 | 1.759 | 0.1206 | -6.222 to 0.7292 |  |
| **IL-2** | 7.048 | 4.296 | 0.1029 | -1.439 to 15.53 |  |
| **IL-6** | 0.2103 | 0.3088 | 0.4968 | -0.3996 to 0.8202 |  |
| **TNF-**α | -0.06569 | 0.2398 | 0.7845 | -0.5394 to 0.4080 |  |
| **BMI** | 0.02317 | 0.1404 | 0.8691 | -0.2542 to 0.3005 |  |
| **Surg. Difficult** | -1.835 | 1.823 | 0.3158 | -5.436 to 1.767 |  |
| **Surg. Duration** | 0.3476 | 0.1272 | **0.007** | 0.09633 to 0.5988 |  |
| **OPMR1** | -3.405 | 1.813 | 0.0622 | -6.985 to 0.1758 |  |
| **COMT** | 2.153 | 1.674 | 0.2003 | -1.153 to 5.459 |  |
| **CPM** | -0.4819 | 1.686 | 0.7754 | -3.812 to 2.848 |  |
| **PCS** | 0.0408 | 0.07049 | 0.5636 | -0.09844 to 0.1800 |  |

Interferon (IFN)-γ, interleukin (IL)-2, (IL)-6, tumor necrosis factor (TNF)-α, body mass index (BMI), opioid receptor (*OPRM1)* and catechol-O-methyltransferase (*COMT)* haplotype, pain modulation capacity (CPM), pain catastrophizing scale (PCS), standard errors (S.E), confidence interval (CI).
