# Supplementary material for: Multifocal Analysis of Acute Pain After Third Molar Removal
Source: Front Pharmacol. 2021 Apr 15;12:643874. doi: 10.3389/fphar.2021.643874 (PMC8082138; doi:10.3389/fphar.2021.643874)
Supplement: Supplementary file 5 [file table5.docx]

**Table S5 –** Multiple logistic regression model. Trismus on the 2^nd^ day after surgery is the dependent variable and interferon (IFN)-γ, interleukin (IL)-2, (IL)-6, tumor necrosis factor (TNF)-α, body mass index (BMI), surgery difficulty and duration, opioid receptor (*OPRM1)* and catechol-O-methyltransferase (*COMT)* haplotype, pain modulation capacity (CPM), and pain catastrophizing scale (PCS) are independent variables

|  | **Trismus** | | | | |
| --- | --- | --- | --- | --- | --- |
| **Variable** | **β** | **S. E** | **P value** | **β - 95% CI** |  |
| **Intercept** | -23.23 | 4.631 | <0.0001 | -32.38 to -14.08 |  |
| **IFN**-γ | 0.3566 | 1.49 | 0.8112 | -2.587 to 3.300 |  |
| **IL-2** | -2.438 | 3.639 | 0.5039 | -9.627 to 4.751 |  |
| **IL-6** | -0.1072 | 0.2615 | 0.6824 | -0.6238 to 0.4094 |  |
| **TNF-**α | 0.1867 | 0.2031 | 0.3594 | -0.2145 to 0.5879 |  |
| **BMI** | 0.2621 | 0.1189 | **0.029** | 0.02717 to 0.4970 |  |
| **Surg. Difficult** | 0.1575 | 1.544 | 0.9189 | -2.893 to 3.208 |  |
| **Surg. Duration** | -0.08231 | 0.1077 | 0.4461 | -0.2951 to 0.1305 |  |
| **OPMR1** | -1.148 | 1.535 | 0.4559 | -4.180 to 1.885 |  |
| **COMT** | -3.606 | 1.418 | **0.0119** | -6.407 to -0.8058 |  |
| **CPM** | 2.67 | 1.428 | 0.0634 | -0.1511 to 5.490 |  |
| **PCS** | -0.09572 | 0.05971 | 0.1109 | -0.2137 to 0.02223 |  |

Interferon (IFN)-γ, interleukin (IL)-2, (IL)-6, tumor necrosis factor (TNF)-α, body mass index (BMI), opioid receptor (*OPRM1)* and catechol-O-methyltransferase (*COMT)* haplotype, pain modulation capacity (CPM), pain catastrophizing scale (PCS), standard errors (S.E), confidence interval (CI).
