# Supplementary material for: Multifocal Analysis of Acute Pain After Third Molar Removal
Source: Front Pharmacol. 2021 Apr 15;12:643874. doi: 10.3389/fphar.2021.643874 (PMC8082138; doi:10.3389/fphar.2021.643874)
Supplement: Supplementary file 6 [file table6.docx]

**Table S6 –** Multiple logistic regression model. Swelling on the 2^nd^ day after surgery is the dependent variable and interferon (IFN)-γ, interleukin (IL)-2, (IL)-6, tumor necrosis factor (TNF)-α, body mass index (BMI), surgery difficulty and duration, opioid receptor (*OPRM1)* and catechol-O-methyltransferase (*COMT)* haplotype, pain modulation capacity (CPM), and pain catastrophizing scale (PCS) are independent variables

|  | **Swelling** | | | | |
| --- | --- | --- | --- | --- | --- |
| **Variable** | **β** | **S. E** | **P value** | **β - 95% CI** |  |
| **Intercept** | -32.47 | 16.26 | 0.0476 | -64.58 to -0.3508 |  |
| **IFN**-γ | -4.421 | 5.232 | 0.3994 | -14.76 to 5.914 |  |
| **IL-2** | 11.36 | 12.78 | 0.3755 | -13.88 to 36.59 |  |
| **IL-6** | 0.4319 | 0.9182 | 0.6388 | -1.382 to 2.246 |  |
| **TNF-**α | -0.2114 | 0.7131 | 0.7673 | -1.620 to 1.197 |  |
| **BMI** | 1.001 | 0.4175 | **0.0177** | 0.1765 to 1.826 |  |
| **Surg. Difficult** | 4.965 | 5.422 | 0.3612 | -5.744 to 15.67 |  |
| **Surg. Duration** | 0.1861 | 0.3783 | 0.6234 | -0.5611 to 0.9333 |  |
| **OPMR1** | -2.55 | 5.39 | 0.6368 | -13.20 to 8.097 |  |
| **COMT** | 4.411 | 4.978 | 0.3769 | -5.421 to 14.24 |  |
| **CPM** | 4.915 | 5.013 | 0.3284 | -4.988 to 14.82 |  |
| **PCS** | 0.3863 | 0.2096 | 0.0673 | -0.02778 to 0.8004 |  |

Interferon (IFN)-γ, interleukin (IL)-2, (IL)-6, tumor necrosis factor (TNF)-α, body mass index (BMI), opioid receptor (*OPRM1)* and catechol-O-methyltransferase (*COMT)* haplotype, pain modulation capacity (CPM), pain catastrophizing scale (PCS), standard errors (S.E), confidence interval (CI).
